# “Lost in translation?” Animal research in the era of precision medicine

**DOI:** 10.1186/s12967-025-06084-3

**Published:** 2025-02-04

**Authors:** Hamideh Frühwein, Norbert W. Paul

**Affiliations:** Institute for History, Philosophy and Ethics of Medicine, University Medical Center, Am Pulverturm 13, 55131 Mainz, Germany

**Keywords:** Murine models, Evidence-based medicine, Precision medicine, 3R, Animal ethics

## Abstract

Preclinical animal research has long been a cornerstone in evaluating the efficacy, toxicity, and safety of potential drug treatments before they proceed to human clinical trials. However, given the intricate nature of human physiology and the complexities of diseases such as cancer, this paper critically examines the role of animal experimentation in translational research, both from epistemological and ethical viewpoints. We argue that the ethical obligation to protect animals extends beyond their instrumental value for human benefit; it is rooted in the intrinsic value of their well-being. Consequently, we advocate for a paradigm shift in medical research: the adoption of new approach methodologies (NAMs) not merely as supplementary tools but as complete replacements for animal use in medical studies. In this context, *replacement* emerges as the key principle—an imperative that should be prioritized over all other considerations.

**Supplementary Information:**

The online version contains supplementary material available at 10.1186/s12967-025-06084-3.

## Introduction

Evidence-based medicine (EBM), first introduced by Sackett and Guyatt in the early 1990s, rests on three core epistemological principles. First [1] it emphasizes the necessity of basing medical practices on the best available evidence, recognizing the varying quality of different evidence types. Second, it advocates for a comprehensive evaluation of all relevant evidence rather than selectively using data that support preexisting beliefs [1]. Finally, EBM acknowledges that evidence alone is not sufficient for decision-making; it is essential to incorporate patient values and preferences, considering the specific context and potential outcomes in clinical decisions [1]. Together, these principles highlight the importance of high-quality, representative evidence in guiding clinical practice.

In this framework, levels of evidence are crucial for prioritizing research findings. Evidence is typically ranked in a hierarchy, with systematic reviews and meta-analyses of randomized controlled trials (RCTs) at the top, followed by individual RCTs, cohort studies, and case-control studies. While higher-level evidence is generally considered more reliable, it is crucial that it not be accepted uncritically. Conversely, lower-level evidence, such as findings from animal studies, should not be dismissed outright but interpreted with care, considering the context and limitations inherent to each type of research.

Animal experimentation has historically played a pivotal role in medical progress, facilitating groundbreaking discoveries such as the germ theory of disease and advancing our understanding of conditions like tuberculosis and cholera. Pioneers like Louis Pasteur (1822–1895) and Robert Koch (1843–1910) relied on animal models to validate their theories and refine medical treatments [2]. The mechanistic view of animals championed by René Descartes during the Renaissance encouraged scientific experimentation, although it also sparked enduring ethical debates about animal suffering and consciousness (Descartes, 1989; Bentham, 1823). Over time, rodents, particularly mice and rats, became the preferred models due to their small size, rapid reproduction, and low maintenance costs, making them ideal subjects for research [3]. In cancer research, knowledge gained from animal research continues to enhance the well-being of not only humans but also domestic, pet, and wild animals [4]. The discovery of antibiotics such as penicillin, erythromycin, and tetracycline would not have been possible without the involvement of animal testing. These advancements highlight the critical role that animal research plays in developing medical treatments that benefit a wide range of species.

However, animal studies, typically regarded as preclinical evidence, occupy the lowest tier in the evidence hierarchy. Although they provide valuable insights, especially in the early stages of scientific inquiry, their translational relevance to human disease is often limited by significant species differences^1^.

A growing body of research highlights the disconnect between preclinical results and human outcomes, underscoring the limitations of animal models as reliable predictors of human disease. For instance, in a systematic review, Henderson et al. [6] identified 26 guidelines and 55 recommendations for designing and executing preclinical efficacy studies to address causal inference threats. Despite recent efforts to enhance preclinical research through data repositories, reporting checklists, biomedical ontologies, and reporting standards, many recommendations remain underutilized [6, 7]. Construct validity threats are also prevalent, as shown by the discrepancy between the time intervals for cardiac arrest and advanced cardiac life support in preclinical versus clinical studies [6]. Henderson et al. noted that while replication studies are sparse, significant replication problems persist in preclinical research. The failure of drugs that pass preclinical trials but fail in human testing underscores the limitations of animal models for clinical translation [6, 8].

The issue of translating preclinical findings into human treatments is particularly evident in cancer research. Despite promising preclinical results, fewer than 15% of clinical trials successfully progress beyond phase I [9], with cancer research consistently exhibiting the highest rates of failure [10]. Even when murine models show initial promise, the success rate of translating these findings into human treatments is less than 8% [9]. The difficulty in translating findings from animal models to human systems underscores the inherent limitations of these models in providing reliable evidence for clinical practice.

Moreover, the rise of personalized medicine (PM) has introduced new complexities in assessing treatment efficacy. PM seeks to tailor treatments to individual patients based on specific genetic, molecular, or biomarker profiles [1]. While this approach offers the promise of more effective, individualized therapies, it challenges traditional methods of evidence generation, such as RCTs and large cohort studies, which may be less applicable when patient populations are small and highly specific. As PM emphasizes the variability of individual responses to treatment, it raises critical questions about the continued relevance of traditional research models like animal studies in the context of modern, personalized approaches to medicine [11].

This context prompts a pressing question: If animal studies are considered the lowest level of evidence, with at least six higher levels available, and if current technological innovations offer viable alternatives to animal use in various stages of research, why do we continue to rely so heavily on animals in scientific inquiry?

In this paper, we further explore the epistemological and ethical concerns surrounding the translation of murine model findings into human clinical applications, in cancer research. The use of murine models has long been a cornerstone of preclinical research, providing invaluable insights into disease mechanisms and potential therapeutic interventions. However, the translation of findings from animal studies, particularly murine models, to successful human clinical applications remains fraught with challenges. Despite their widespread use, the low success rates of translating murine research into effective human therapies raise pressing questions about the reliability and validity of these models in the mode of EBM. Moreover, the emergence of PM, which emphasizes individualized patient care based on genetic, environmental, and lifestyle factors, has further highlighted the limitations of traditional animal models. As precision and personalized medicine redefine clinical practice, the adequacy of these models to capture the complexity of human diseases is increasingly being questioned.

Against this backdrop, ethical considerations surrounding animal experimentation have also intensified. Balancing the scientific benefits of animal research with the moral imperative to minimize harm to animals adds another layer of complexity to this discourse. These epistemological and ethical concerns call for a reevaluation of the role and hierarchy of evidence in EBM. In this context, this study seeks to address the question: How can the epistemological and ethical challenges of translating murine model findings into human clinical applications inform the development of alternative approaches in evidence-based medicine, particularly in the context of precision and personalized medicine?

### Epistemological perspective: evidence and animals

A pivotal critique of animal research came in 2004 with the publication of a commentary in the *British Medical Journal* titled “Where is the evidence that animal research benefits humans?” In this study [12], researchers conducted a meta-analysis of six systematic reviews, reported a series of issues with animal studies and reports, and questioned the validity of evidence presented by such research. They noted a lack of methodological rigor, including improper randomization and flawed experimental designs that undermined the reliability of the results. In some cases, animal studies were conducted in parallel with clinical trials, distorting the sequence of research and undermining the role of animal models as proof-of-concept before advancing to human trials. Additionally, the small sample sizes in many studies led to inconclusive results, making it difficult to justify their use in clinical trials. Pound and colleagues’ [12] review concluded that animal research often fails to provide conclusive evidence of its predictive value for human outcomes. Further scrutiny comes from Lieu et al. [13], who analyzed translational research in cancer drug development. They reviewed numerous examples of negative phase III trials and assessed studies related to basic cellular processes, angiogenesis, and targeted therapies against oncogenic drivers. They concluded that the primary reasons for failure include a lack of efficacy and clinically significant toxicity. Moreover, they noted that the predictive value of preclinical models is often limited by factors such as individual genetic sensitivities, immunologically mediated phenomena, and idiosyncratic reactions [13]. Additional criticisms of animal studies include a lack of clear objectives and hypotheses [14], as well as publication bias, with negative results frequently remaining unpublished, leading to an overestimation of treatment effectiveness by approximately 30% [15].

The Reproducibility Project: Cancer Biology (RPCB), initiated in 2014 by the Center for Open Science and Science Exchange, aimed to address these concerns by assessing the replicability of preclinical cancer research. This project was spurred by claims from Bayer and Amgen that they could not replicate most cancer research results. The RPCB team selected 193 experiments from 53 high-profile papers published between 2010 and 2012. However, they encountered significant challenges, including insufficient methodological details and a lack of statistical transparency in the original papers. Consequently, they could only replicate 50 experiments from 23 papers. The RPCB report emphasized that their goal was not to critique specific experiments but to encourage biomedical researchers to reassess the reliability of their results [16]. Additionally, Hay et al. [17] reported a troubling failure rate of 19 out of 20 for cancer drugs that showed promising results in preclinical phases but failed in clinical trials owing to inefficacy. These failures highlight the methodological shortcomings prevalent in cancer research [18]. Researchers [8] report the same pattern of unreliability and unreplicability in spinal cord injury, stroke, Parkinson’s disease and multiple sclerosis. Furthermore, in cases like Alzheimer’s disease, over 99% of clinical trials fail, with no animal model reliably predicting the clinical effectiveness of Alzheimer’s treatments [19].

Additionally, although it has been argued [20] that information derived from animal experiments is better than no information at all, this perspective overlooks the risks of misleading data, which can be more harmful than no data. Non-predictive animal experiments can cause human suffering in two main ways: [1] by producing inaccurate safety and efficacy data, and [2] by diverting resources away from more effective testing methods and potentially abandoning beneficial treatments [20]. Misleading animal test results may lead to clinical trials of biologically flawed or harmful substances, exposing patients to unnecessary risks and wasting valuable research resources. Particularly, animal toxicity studies have been shown to be poor predictors of human drug toxicity. As a result, clinical trial participants may be misled into a false sense of security regarding the safety and efficacy of treatments based on animal testing.

In this context, an additional issue warrants attention: the ethical and practical implications of animals being bred for scientific research purposes but ultimately not utilized in experiments. In their paper “Bred, but not used”: Understandings of Avoidable and Unavoidable Waste in Animal Research, Sara Peres and Emma Roe [21] examine the ethical and practical challenges associated with animal research, particularly focusing on the notions of avoidable and unavoidable waste. They highlight the fact that many animals are bred specifically for research but ultimately never used in experiments, raising critical ethical questions about the implications of such practices. Peres and Roe explore how this phenomenon reflects broader issues related to resource allocation, the value placed on animal lives, and the moral responsibilities of researchers within the context of scientific inquiry. The authors argue that these practices suggest a need to reconsider the ethical frameworks that guide animal research, with an emphasis on minimizing waste and ensuring more humane treatment of animals. As Peres and Roe suggest, the value of animals is not solely determined by how they are cared for, but also by the degree to which their lives are valued. This perspective highlights the complex and multifaceted ways in which animals are regarded within the scientific community. By adopting a diverse economies approach, the authors demonstrate that animals hold significance that extends far beyond their functional role as research subjects. This broader valuation challenges the reductive view of animals as mere tools for scientific progress and encourages a more ethical and responsible approach to animal research (we will circle back to this point later in the discussion).

The emergence of PM has further complicated the role of traditional research models like animal studies. PM critiques the assumptions underlying randomized trials, particularly the idea that individuals respond similarly to the same treatment [22]. De Leon [22] contrasts PM with EBM, highlighting that EBM often assumes that drug responses are uniformly predictable, using average responses as a proxy for individual outcomes. In contrast, PM emphasizes that such uniformity is unrealistic, arguing instead that responses to treatments are inherently varied across different individuals.

The use of animals in N-of-one trials exemplifies the complexities and challenges inherent in precision medicine [23], particularly in cancer research. These trials, which focus on tailoring treatments to individual patients, often involve intermediary animal models such as patient-derived xenografts (PDX). In this process, a patient’s tumor undergoes detailed molecular analysis to identify specific drivers, and potential therapies are tested on the tumor samples implanted in mice. While this approach aims to identify effective treatments, it raises ethical concerns about the necessity and harm caused to animals, especially when alternative models like organoids or computational methods may offer comparable insights. The reliance on animal models is also questioned concerning the variability in therapeutic responses [24], as even patients sharing a biomarker may exhibit inconsistent outcomes due to differences in tissue types, nongenomic factors, and the tumor microenvironment. Adding to this complexity, cancer biology is influenced by dynamic and multifaceted molecular processes, including spatial and temporal variations in gene expression and tissue-specific factors [23], which significantly impact treatment efficacy. These challenges highlight a broader limitation in precision medicine, where the focus on molecular-level data often fails to address the higher-order biological variability that shapes disease progression and treatment outcomes. Together, these issues call for a critical reevaluation of current practices, emphasizing the need for more ethically sound and scientifically robust alternatives that better capture the intricacies of human disease.

Given the epistemological challenges surrounding the translation of animal research to human clinical outcomes, this article furthermore aims to explore the role of ethics in the relevance and validity of animal models in translational research. By critically examining both the scientific and ethical dimensions, we seek to highlight the need for a more nuanced approach to the use of animal models in medical research, one that considers both the limitations of current practices and the promise of alternative methodologies.

### Ethical perspective: evidence and animals

The philosophical critiques of animal use in research offered by Peter Singer, Martha Nussbaum, Josephine Donovan, Gary Francione, Tom Regan, and Christine Korsgaard – among others- provide robust ethical frameworks for opposing the exploitation of animals in scientific contexts. These critiques challenge the underlying assumptions that justify animal experimentation, particularly the anthropocentric and speciesist beliefs that position human lives as inherently more valuable than those of animals. By examining the moral status of animals, the capacity for suffering, and the principles of justice, these philosophers collectively argue against animal research, advocating for the recognition of animals as sentient beings with intrinsic moral worth, independent of their utility to humans.

Peter Singer [25], a leading (preference-) utilitarian philosopher, argues that the capacity to suffer, rather than species membership, should be the basis for moral consideration. In his influential work, *Animal Liberation* (1975), Singer contends that all sentient beings—humans and nonhumans alike—are entitled to equal moral consideration. This principle, which he calls the *principle of equal consideration of interests*, rejects the idea that the suffering of animals can be dismissed simply because they are nonhuman. In the context of animal research, Singer asserts that causing animals unnecessary pain, distress, or death is ethically indefensible unless it leads to significant benefits for the greater good, and even then, only if the benefits clearly outweigh the harms. Given the availability of non-animal research alternatives, Singer’s philosophy would advocate for the reduction of animal experimentation and the pursuit of more humane and effective research methodologies as he has formulated in his most recent edition *Animal Liberation Now (2023)*. Similarly, Martha Nussbaum’s *capabilities approach* [26] provides a robust framework for assessing justice for both human and nonhuman animals. Nussbaum argues that a just society must ensure that all beings—human or nonhuman—have the fair opportunity to live a life in which they can flourish, in accordance with their species-specific potential. Nussbaum’s theory focuses not only on preventing suffering but also on promoting the conditions necessary for beings to live fulfilling lives. In relation to animal research, Nussbaum comments on practices that deprive animals of their ability to live in accordance with their natural capabilities, such as their capacity for health, social interaction, and autonomy. Research that subjects animals to confinement, stress, and pain impedes their ability to thrive, which, according to Nussbaum, violates principles of justice. Therefore, animal experimentation must be reimagined as a practice that respects animals’ flourishing and ensures that alternatives are pursued whenever possible.

Josephine Donovan [27, 28], drawing from feminist ethics of care, emphasizes the relational nature of moral duties. From this perspective, ethics is not about abstract rules but about understanding and responding to the specific needs and vulnerabilities of others within a context of care. Donovan argues that moral consideration should extend to all beings with whom we share a relational bond, and that this bond is inherently disrupted when animals are treated as mere resources or tools for human exploitation. In the context of animal research, Donovan analyzes the objectification of animals as experimental subjects, calling for a more empathetic and contextually sensitive approach to our ethical obligations to them. From a feminist care ethics standpoint, animal research is ethically troubling because it ignores the individual interests and well-being of animals, reducing them to mere means to human ends.

More importantly for our argument here, Gary Francione’s [29] abolitionist stance builds on the idea that animals have intrinsic value and should not be exploited for human benefit. Francione’s animal rights theory rejects *speciesism*, the idea that humans have greater moral worth than nonhumans solely based on their species membership. Francione maintains that animals, as sentient beings, have inherent rights—most fundamentally, the right to not be treated as mere property or instruments for human gain. In the context of animal research, Francione argues that no form of animal experimentation is morally justifiable, as it inherently violates the rights of animals by treating them as mere tools for human ends. Furthermore, he emphasizes that alternatives to animal research are available, and that the development of such alternatives should be prioritized. For Francione, the abolition of animal experimentation is not just a moral imperative but a necessary step toward a society that respects the intrinsic value of all sentient beings. This is indeed akin to Tom Regan’s rights-based approach to animal ethics [30] which builds on the notion that animals, as *subjects-of-a-life*, possess inherent moral worth. Regan argues that, like humans, animals have interests that matter to them, and these interests must be respected. According to Regan, the fundamental moral principle is that individuals should never be treated as mere means to an end, but always as ends in themselves. This principle is central to his critique of animal research. Regan asserts that the use of animals in experiments—where they are often subjected to pain, confinement, and death—is an explicit violation of their basic rights. In his view, the moral status of animals does not depend on their potential utility to humans but on their intrinsic value as sentient beings. Thus, Regan advocates for the abolition of animal experimentation, calling for a radical shift in how we approach our ethical obligations to animals in scientific research.

Christine Korsgaard [31], interpreting Kantian ethics, expands the moral community to include all sentient beings, arguing that moral duties are owed to nonhuman animals because they are *subjects of a life*—beings with experiences that matter to them. While Kantian ethics traditionally excluded animals from moral consideration based on their lack of rational agency, Korsgaard modifies this by asserting that all sentient creatures, regardless of their cognitive capacities, have interests that merit moral respect. For Korsgaard, the moral obligation to respect the autonomy and dignity of animals requires that their rights be honored in all contexts, including scientific research. In her view, animal experimentation cannot be morally justified, particularly given the availability of alternative research methods that do not involve the exploitation of animals.

These philosophers challenge the justifications often made for using animals in research, calling for a more ethical and humane approach that respects the intrinsic value of animals, minimizes suffering, and advocates for alternative research methods. Below is an integration of their views with the role of the 3Rs (refinement, reduction, replacement) in addressing ethical concerns:

1. Intrinsic Moral Value of Animals: Philosophers like Francione, Regan, and Korsgaard emphasize that animals, as sentient beings, possess intrinsic moral value that must be respected. From a deontological perspective, treating animals as mere tools or commodities in research fundamentally disrespects their inherent worth. This view calls into question the ethical justification for using animals in experiments where their moral consideration is secondary to human interests. In this light, the 3Rs, particularly replacement and reduction, serve as essential strategies to uphold the intrinsic moral value of animals. By prioritizing non-animal alternatives and reducing the number of animals used, researchers can avoid the exploitation and objectification of animals, aligning research practices more closely with the moral respect that animals deserve.
2. Suffering and Welfare: Singer and Nussbaum highlight that inflicting suffering on animals in research is ethically problematic. Singer, a preference utilitarian, argues that the capacity to suffer should be a central criterion in moral consideration, urging that we minimize suffering wherever possible. Nussbaum’s capabilities approach further underscores that research practices that harm animals undermine their ability to flourish as sentient beings. Both perspectives make a strong case for reducing animal suffering in research settings. Here, refinement and reduction are highlighted. Refinement seeks to improve experimental protocols to reduce pain, distress, and unnecessary suffering, while reduction emphasizes using fewer animals, thus minimizing the overall impact on animal welfare.
3. Alternative Methods: The call for alternatives to animal testing is a central theme for philosophers such as Francione, Regan, and Korsgaard, who argue that science can and should progress without exploiting animals. These thinkers stress that non-animal methods are increasingly available and should be prioritized, pushing for innovation in alternative research methodologies. The 3Rs directly support this ethical framework by advocating for replacement. This principle aligns with the growing body of research into alternatives such as organ-on-a-chip technologies, in vitro models, and computer simulations.
4. Moral Agency and Moral Obligations: Philosophers like Korsgaard and Donovan emphasize that humans have a moral obligation to care for animals, rather than exploit them for research purposes. They argue that humans must recognize the relational responsibilities we have toward animals and reject their objectification. Korsgaard, in particular, insists that humans, as moral agents, have a duty to treat animals with respect, taking into account their well-being and ensuring that their treatment aligns with ethical standards. Reduction and Refinement can be viewed as part of the broader ethical duty to respect animals’ interests, reduce unnecessary harm, and refine research practices to limit suffering. By promoting practices that align with Korsgaard’s view of relational responsibilities and Donovan’s emphasis on moral obligations, the 3Rs provide a practical approach for ethically engaged researchers to minimize harm and recognize animals as moral subjects.

In sum, the integration of ethical theories with the principles of the 3Rs—replacement, reduction, and refinement—provides a robust framework for addressing the moral concerns surrounding animal research while reconciling philosophical perspectives with practical scientific realities. Utilitarianism emphasizes minimizing suffering and maximizing welfare, aligning with efforts to reduce harm and adopt alternatives to animal use. Deontological ethics underscores the intrinsic moral worth of animals and the duty to respect their rights, which is supported by the replacement and reduction of animals in research. Virtue ethics, particularly Martha Nussbaum’s capabilities approach, calls for the promotion of justice and flourishing for all beings, reinforcing the need to refine research practices to respect animals’ capacities. Feminist care ethics adds an empathetic perspective, advocating for relational responsibilities and practices that minimize distress, in line with refinement. Rights-based ethics categorically opposes the exploitation of animals, supporting the complete replacement of animal models with alternatives. While these ethical frameworks may diverge in their broader commitments, they converge on the moral imperative to reduce harm, respect animals’ intrinsic value, and prioritize humane and innovative methodologies. The 3Rs thus provide a practical pathway for aligning scientific practices with these ethical principles, emphasizing that animals should not be subjected to unnecessary harm and that alternative methods must be prioritized wherever feasible. Together, they foster a more compassionate and morally responsible approach to research.

### Paradigm to be altered

To build our argument now, it is essential to revisit the historical and ethical contexts surrounding animal research and its alternatives. On the one hand is Rudolf Virchow. In 1845, working at the Charite ´Hospital in Berlin as a military Surgeon Rudolf Virchow was asked to give a speech at a celebration of the birthday of the founder of the institute. This was entitled ‘Über das Beduerfnis und die Richtigkeit einer Medizin vom mechanischen Standpunkte’ [On the need and correctness of a Medicine based on a mechanistic approach]. In this address, Virchow outlined three core principles he deemed essential for medical progress: clinical observation, animal experimentation to understand disease mechanisms and test drugs, and pathological anatomy, particularly at the microscopic level. Virchow’s emphasis on the cell as the fundamental unit of life underscored the necessity of studying its structure and function to understand and treat diseases [32]. These principles were mirrored in his co-founding of the journal Archiv für pathologische Anatomie und Physiologie und für klinische Medizin (Archives for Pathological Anatomy and Physiology and Clinical Medicine) in 1846, which exclusively published evidence-based, rigorously tested medical findings. Virchow’s research on pulmonary thrombi, conducted through meticulously designed and repeated experiments on dogs, exemplified his commitment to empirical validation as a means to improve medical practice for the broader population, especially the socially underprivileged [32]. However, such principles have become a focal point of ethical criticism, as addressed in earlier sections.^2^

On the other hand, there is the 3Rs principle. While the 3Rs have long served as the ethical framework for evaluating the use of animals in scientific research, the rapid advancement of non-animal research technologies such as Alternative New Methodologies (NAMs) calls for a fundamental reevaluation of this approach. As we have shown in the previous section, historically, the 3Rs have sought to minimize harm and improve the welfare of animals used in research, yet these principles are increasingly insufficient given the availability of sophisticated alternatives. In practice, the ethical justification for animal research is primarily based on harm–benefit analysis (HBA) [33], which weighs the potential benefits of the research against the harm caused to animals. This approach is reflected in key documents like the EU Directive 2010/63 and the US National Research Council’s *Guide for the Care and Use of Laboratory Animals* (8th edition). The regulatory framework ensures that animal use is only approved when the benefits are clear and substantial, while efforts are made to minimize harm.

Against this background, we propose a paradigm shift from the 3Rs to an exclusive focus on *replacement*. This shift is not merely a call for reducing the number of animals used in research, nor is it a plea for refining experimental techniques to cause less suffering. Rather, it is a recognition that, with the advent of cutting-edge technologies such as organ-on-chip models, 3D cell cultures, advanced computational models, and artificial intelligence-driven simulations, animal research may no longer be scientifically necessary or ethically defensible. These alternatives offer more accurate, relevant, and humane ways to conduct scientific investigations, rendering the use of animals in research increasingly obsolete.

The argument for replacement rather than reduction or refinement is grounded not only in the moral obligation to recognize animals as sentient beings with intrinsic value but also in the growing scientific reality that animal testing is no longer the most effective or reliable method. From the perspective of philosophers like Regan and Francione, animals possess inherent rights and should not be treated as tools for human gain, regardless of how much harm may be minimized. Nussbaum’s capabilities approach reinforces this argument, emphasizing that justice demands we create conditions where animals can thrive, not merely endure less harm. In this context, replacement becomes not just a moral imperative but a scientific one: given that non-animal models are now capable of providing more accurate and human-relevant data, animal testing can be seen as not only unethical but unnecessary.

Thus, the ethical focus should no longer be on reducing animal suffering through the 3Rs, but on completely eliminating animal use in research. This shift would signal the recognition that the moral obligation to protect animals is inextricably linked to our ability to develop and utilize technologies that do not involve animal suffering. By focusing exclusively on replacement, we acknowledge that we have reached a point where animal testing is no longer acceptable, either scientifically or ethically.

The future of research could be one in which animals are no longer subjects of experimentation but are instead respected as beings with intrinsic moral worth, and their use in science is replaced entirely by innovative, more humane alternatives.

Martha Nussbaum’s [26] perspective on animal ethics aligns with this paradigm shift. In her critique of utilitarianism, Nussbaum argues that ethical frameworks must start with a Kantian respect for sentient beings. According to Nussbaum, we must respect each individual sentient being as an end in itself, not as a mere means to the ends of others [26]. This approach challenges the utilitarian focus on balancing suffering against benefits, a stance that underpins the 3Rs, which justify animal testing as long as harm is minimized and benefits maximized. Nussbaum asserts that the lives of animals have intrinsic value—this value is independent of human choice or legislation, meaning animals’ lives are valuable whether or not humans exist to acknowledge them [26]. If a scientific procedure can be conducted without the use of animals, such as through in vitro testing, computational models, or human cell cultures, replacing animal testing aligns with Nussbaum’s principles of justice and fairness [34]. Justice, she contends, requires recognizing the moral subjecthood of animals and upholding their rights to live free from unnecessary harm, thus rejecting the view that animal use is permissible unless absolutely essential.

Nussbaum’s formulation of justice requires recognizing that animals, as sentient beings, should not be treated as mere resources or instruments for human use. Instead, they should be seen as ends in themselves, deserving of respect and protection, regardless of human utility. In her framework, the recognition of animals’ intrinsic value leads us to a moral duty to pursue non-animal research methods that can fulfill scientific needs without inflicting harm.

From the perspective of utilitarianism, animal testing has historically been justified by weighing the benefits of scientific progress against the harms inflicted on animals. The 3Rs are a direct reflection of the utilitarian approach, aiming to minimize animal suffering while still allowing scientific research to proceed for the greater good. However, this framework falls short in addressing the moral status of animals as individuals. As Nussbaum points out, justice is not about reducing harm to animals [34]; it is about ensuring that animals have the right to live lives free from unnecessary suffering, in accordance with their intrinsic worth.

In contrast to utilitarianism, the Kantian and neo-Aristotelian elements of Nussbaum’s approach argue that animals, like humans, possess inherent value that must be respected, not merely treated as a means to an end. Korsgaard, whose work Nussbaum draws on, also emphasizes that animals are ends in themselves, deserving of moral consideration and care. Korsgaard argues that we should treat animals in ways that honor their complex lives and their self-maintaining systems, recognizing their intelligence and the richness of their existence [26]. This aligns with our proposal to replace animal testing with more humane alternatives. The moral and scientific landscape has changed. Animal testing is no longer necessary to advance science, and its ethical justification is increasingly untenable.

Ethically, human lives are generally prioritized over nonhuman entities, resulting in greater perceived responsibility for human health. However, in the past two decades, improvements have been made largely due to the introduction of standardizations and control procedures in drug design and testing [35]. These advancements have been driven by alternative methodologies that are increasingly used in basic research. However, as researchers [35] observe, the application of these alternative methods is often limited to the laboratories where they were initially developed, pointing to a pressing need for broader technology transfer.

The establishment of living biobanks, which store cryopreserved organoids for research and drug screening, represents a significant step forward in medical research, particularly in cancer treatment. These biobanks facilitate access to tumor samples that capture the heterogeneity of cancer, thus enabling more precise and effective drug development [36]. Crucially, the development of organoids—miniature, human-like organ structures grown from stem cells—also introduces a profound ethical consideration regarding the use of animals in research. As potential alternatives to traditional animal models, organoids offer the opportunity to advance medical research while minimizing animal suffering. From a contractarian perspective, the promotion of such alternatives aligns with the principle that humans, as rational beings, have an ethical obligation to protect animal welfare through technological innovation. The use of organoids, therefore, embodies a more ethically responsible approach, where both human and animal interests are balanced within a framework of mutual benefit. If organoids can replace animals in certain areas of research, it would fulfill a kind of “social contract” that reduces harm to animals while continuing to drive scientific progress for the benefit of human society. As noted by Green et al. [36], organoids are emerging as superior translational models for cancer research, because they offer the ability to test a broader range of cancer subtypes, which is crucial for personalized medicine. Innovative scientific strategies that center on human biological systems to study diseases and assess potential treatments-collectively referred to as NAMs- utilize human cells, tissues, organs, and existing data, and incorporate advanced technologies such as human cell cultures, organ-on-a-chip models, and artificial intelligence [37]. Since NAMs focus specifically on human biology, they avoid the challenges of translating findings from one species to another, offering results that are more directly applicable to human patients [38]. During the COVID-19 pandemic, NAMs demonstrated their value in drug discovery [38].

Elsewhere Pun and colleagues [39] emphasize the considerable progress achieved through innovations in stem cells, organoids, organ-on-chip technologies, and 3D bioprinting. These developments have enabled the design and fabrication of living organ biomimetic systems that more accurately replicate human tissue function and structure. These systems are increasingly used to predict human responses to drugs and environmental stimuli, offering an ethical and potentially more accurate alternative to traditional animal testing. The concept of culturing cells and tissues in a controlled microenvironment to replicate cell and tissue function in vitro has been a cornerstone of biomedical research for over a century, but only with recent technological advancements are these methods beginning to fulfill their potential as replacements for animal models.

## Discussion

Since the introduction of the 3Rs, numerous initiatives have been established to promote alternative research methods. In 1969, the Fund for the Replacement of Animals in Medical Experiments (FRAME) was founded, followed by the journal Alternatives to Laboratory Animals (ATLA). In 1981, the Center for Alternatives to Animal Testing (CAAT) was created at Johns Hopkins University, and the European Reference Laboratory for Alternatives to Animal Testing (EURL-ECVAM) was founded in 1991 to enhance the acceptance of non-animal methods. In 2000, the U.S. Interagency Coordinating Committee on the Validation of Alternative Methods (ICCVAM) was established to validate alternative toxicological tests. The Council for International Organizations of Medical Sciences (CIOMS) published updated guiding principles in 2012, and the ISO 10993-2 standard was introduced in 1996 to incorporate animal welfare. Additionally, the World Animal Health Organization (OIE) included animal welfare standards in its Terrestrial Animal Health Code. These initiatives reflect a global shift towards reducing animal testing and advancing humane research practices [40].

Significant progress has already been made by the scientific community, industry, and regulatory bodies in this area. The novel approaches discussed—incorporating cutting-edge computational technologies, bioengineering innovations, and big data analytics—are not entirely new. These methods are actively being developed to reduce the reliance on animal models in cancer research and treatment development and are increasingly being integrated into mainstream cancer care strategies. Key examples include AI-driven cancer models for drug discovery [41], human microbiome models in cancer research [42], patient-derived tumor organoids [43], crowdsourced cancer data for virtual trials [44], CRISPR-based humanized models for targeted cancer therapy [45], microfluidic-based screening [46], synthetic biology for cancer research [47], and decentralized clinical trials using blockchain technology [48]. These approaches represent a convergence of interdisciplinary strategies designed to create more accurate, human-relevant, and ethical models for cancer research, thereby advancing the broader goal of animal reduction. Furthermore, the European Union’s ban on animal testing for cosmetics (effective from 2013) exemplifies a deontological response to the ethical concerns raised by animal rights activists. The ban reflects a growing societal recognition that animals should not be subjected to unnecessary suffering for non-essential purposes, such as the production of cosmetic products.

That said, operationalizing these advances remains a significant challenge. Despite the myriad promising alternatives that adhere to the 3Rs principles, we are still, to some degree, dependent on animal studies, particularly in certain stages of research or in contexts where human-relevant models have not yet fully matured [49].

Historically, arguments for animal research centered around several main points: (a) human life was considered to take precedence over non-human life, making animal testing acceptable if it advanced human health; (b) lifesaving breakthroughs like penicillin and vaccines were attributed to animal research, reinforcing its perceived necessity; (c) the lack of alternatives, especially in earlier centuries, meant the choice was often between animal or human testing, with animals ultimately deemed the more ethical option; and (d) the belief that animal biology sufficiently resembled human biology, justifying their use in toxicity and efficacy tests. However, recent advances challenge each of these rationales: for example, the COVID-19 vaccine was developed using AI and new in vitro technologies, illustrating that breakthroughs are now possible without animal involvement.

Considering the identified challenges with animal models in translational research and the evolving landscape of personalized medicine, we propose several practical solutions to address epistemological and ethical issues while enhancing the applicability and effectiveness of evidence-based medicine.

1. Prioritizing the development and use of NAMs: Advances in stem cell technology, organoids, and organ-on-chip systems present promising alternatives that closely replicate human biology and pathology [39]. Research institutions and funding agencies are encouraged to allocate resources and support for the development and application of these technologies. Furthermore, updating regulatory frameworks to recognize and validate these alternative models will facilitate their broader adoption in preclinical research. The VCI’s (German Chemical Industry Association [37]) position paper on NAMs advocates for the expansion and integration of innovative testing methods that aim to replace or reduce animal testing. NAMs include in silico models, in vitro assays, and other high-throughput tools like genomics and proteomics. The paper emphasizes that these methodologies are crucial for advancing scientific understanding while improving safety assessments for chemicals. The VCI calls for regulatory support and recognition of these methods to ensure their broader application in industry and research. The use of NAMs, we need to point out, has been more difficult.

For example, some scholars [50] who explored the potential of alternative research methodologies, such as computational models, organ-on-a-chip systems, and in vitro techniques, to replace animal testing, maintain that while these methods hold promise for more accurate and humane science, their widespread adoption faces several obstacles, including regulatory challenges and the need for rigorous scientific validation. The authors stress the importance of collaboration between academia, industry, and regulatory agencies to overcome these barriers and accelerate the transition to NAMs in scientific practice.

These scholars [50] make it clear that NAMs are not intended to be direct replacements but provide more relevant and humane ways of assessing chemical safety, particularly for systemic toxicities such as carcinogenicity and reproductive toxicity by emphasizing that NAMs are conceptually different from animal testing, focusing on human-relevant data rather than replicating animal responses. The article critiques the benchmarking of NAMs against animal models, such as rodents, which have shown limited predictive value for human toxicity. Overall, while NAMs offer a more human-focused approach, their integration into safety assessments requires advances in exposure science and robust validation methods.

Elsewhere [51] NAMs has been clearly defined as “any technology, methodology, approach, or combination that can provide information on chemical hazard and risk assessment without the use of animals, including in silico, in chemico, in vitro, and ex vivo approaches” particularly “it is their application to regulatory decision making or replacement of a conventional testing requirement that is new”.

However, they [51] also highlight that:

“In October 2020, the EU Chemicals Strategy for Sustainability (CSS) Towards a Toxic-Free Environment was published (EC, 2020). It identified a need to innovate safety testing and chemical risk assessment *to reduce dependency on animal testing* while improving the quality, efficiency, and speed of chemical hazard and risk assessments. However, fulfilling its additional information requirements will *more likely lead to an increase in animals used*. Also, it is currently unknown whether the implementation of the CSS will open opportunities for the application of more NAMs” (p.5 our emphasis).

The issue is further complicated then by CSS, which stresses the need to innovate safety testing but also warns that fulfilling additional data requirements may lead to an increase in animal testing. Stucki et al. [51] express concern about whether the CSS will genuinely open opportunities for NAMs or simply reinforce traditional methods. Thus, while NAMs have the potential to revolutionize chemical safety assessments, their adoption will require overcoming significant scientific, technical, and regulatory hurdles, as well as changing long-standing practices in risk assessment. However, in 2021, the U.S. Environmental Protection Agency (EPA) allowed in vitro testing on human skin for agrochemical formulations, which was shown to be equally or more protective than animal-based methods [51]. Additionally, in chemico testing was used to assess a polymer’s potential to cause lung overload, resulting in the revocation of a previous animal testing requirement after the in chemico data confirmed no hazard ([51]). These examples are consistent with broader global efforts to move away from animal testing and embrace more advanced, human-relevant methodologies.

2. Implementing and strengthening personalized medicine approaches: Personalized medicine requires a departure from conventional clinical trial designs toward methodologies that cater to individual patient profiles. The following strategies should be considered:

- N-of-One Trials: Implementing N-of-one trials allows for the tailoring of treatment decisions on the basis of individual patient characteristics. This approach, which aligns with the principles of PM, provides personalized treatment regimens informed by unique genetic and molecular profiles. Ensuring rigorous statistical methods and comprehensive reporting in these trials will increase their scientific validity and relevance [52].
- Precision oncology: Addressing the complexities of cancer biology through innovative trial designs is crucial. Adaptive trial designs and Bayesian approaches, which offer flexibility in assessing treatment efficacy, should be adopted to better address the variability of cancer responses [23]. Integrating these designs into standard practice will improve the assessment of personalized cancer therapies.

## Conclusion

In 2016, researchers [33] working on the concept of harm-benefit analysis of animal experiments, wrote that “Historical success of animal experimentation is not sufficient to justify continued animal use, as science is constantly evolving and alternative methods can become available”. Today, we can already say that alternative methods (NAMs for example) *have* become available. Today, advancements in NAMs have demonstrated a credible path forward in many areas of research. Scholars have pointed out that, while animals differ in ways that undermine the reliability of data extrapolation to humans, they share a capacity for suffering, making animal testing ethically questionable and scientifically unreliable [38].

The traditional 3Rs principles of replacement, reduction, and refinement, intended to ethically guide animal testing, has in practice often served to reinforce its continued use rather than to eliminate it. This principle, as critics like Nussbaum observe, can reflect a Kantian view, where animals are seen as means to human ends rather than beings with intrinsic worth [26]. Even with the 3Rs in place, animals are still subjected to harm, as regulatory frameworks justify their use based on indirect duties to humans rather than direct moral obligations to the animals themselves.

The development of organoid-based testing and other NAMs highlight a possible shift, where agencies like the FDA might soon validate these technologies as replacements for animal testing, not just supplements. Researchers like Green, Dam, and Svendsen [36] have advocated for regulatory acceptance of NAMs, anticipating they will become an integral part of human-relevant, ethically conscious drug development. With NAMs continually proving their scientific reliability and ethical value, there is an opportunity to fully embrace replacement over reduction and refinement.

The time has come for a decisive shift away from animal testing and toward technologies that prioritize accuracy, facilitate precision medicine and establish more humane research practices. By decisively adopting NAMs, the scientific community can reflect a commitment to both animal welfare and improved research methodologies. This approach is not only ethically necessary but also promises to enhance the validity and translatability of scientific outcomes, signifying a new era where progress in research and ethics coalesce, establishing a responsible, animal-free standard in scientific discovery.

## Electronic supplementary material

Below is the link to the electronic supplementary material.

Supplementary Material 1

## Data Availability

Not applicable.

## Footnotes

1 For a critic on how the mere Pyramid-shaped l representation of evidence see Blunt [5] who has reviewed the evidence pyramid, its origins and impact on the meaning of evidence.

2 For example, toxicity testing for new vaccines has been determined via a mandatory experiment on animals (Mice and Guiana pigs) where 2 guinea pigs and 5 mice are injected with the product being tested. If the animals survived symptom-free for 7 days, the vaccine (or the new drug) was declared safe for huams [33]. However, upon their investigation, Behrensdorf-Nicol and Krämer (2014) found out that this test is more than a century old and was originally used for diphtheria and tetanus sera. Although FDA and WHO demanded the drug industry to present the result of this test for every newly developed drug since 1940s, in 1996, Rajesh K. Gupta proposed the test to be eliminated from vaccine safety studies. This was not only because it was incompatible for its purposes but also to reduce animal suffering, and in 1997 it was deleted from almost all monographs of the European Pharmacopoeia without replacement [34].

## Declarations

### What the article reports

The article critiques the dominant use of animal testing in scientific research, arguing that the historical justification, rooted in figures like Rudolf Virchow’s 19th-century principles, is now outdated due to advancements in non-animal methodologies such as organoids, 3D cultures, and computational models. These technologies offer scientifically robust and ethically viable alternatives, challenging the adequacy of the 3Rs (Reduction, Refinement, Replacement) framework. Citing ethical theories from Nussbaum and Korsgaard, the article argues for a paradigm shift that values animals as beings with intrinsic worth, rather than instruments for human benefit, suggesting that the moral and scientific imperatives for replacement are stronger than ever.
