# Supplementary material for: “Lost in translation?” Animal research in the era of precision medicine
Source: J Transl Med. 2025 Feb 4;23:152. doi: 10.1186/s12967-025-06084-3 (PMC11796152; doi:10.1186/s12967-025-06084-3)
Supplement: Supplementary file 1 — Supplementary Material 1 [file 12967_2025_6084_MOESM1_ESM.docx]

Reviewer #1: The manuscript titled "Lost in Translation? Animal Research in the Era of Precision Medicine" provides an insightful and well-structured exploration of the challenges surrounding animal research, particularly within the context of cancer and precision medicine. The authors highlight significant concerns regarding the translatability of animal models to human clinical applications, emphasizing both epistemological and ethical perspectives. The paper was well written
The manuscript presents a well-rounded review of the current challenges with animal research, particularly in cancer, in the era of precision medicine. The authors accurately reference key studies and meta-analyses that critique the translational success of animal models. The discussion is detailed, well-sourced, and covers important literature in both cancer research and the epistemological problems of animal studies. The conclusion is well-structured and presents several practical solutions aimed at addressing the ethical challenges.
Few points to consider.

*1. The authors overemphasize that the challenges of translatability of animal models to human applications misalign with the principles of EBM and affect "the reliability and applicability of preclinical animal research in informing clinical decisions, thereby necessitating a critical examination of its role in evidence-based medicine". The authors appropriately describe the challenges in this translation; however, they miss to point out that evidence based on bench research is level 5, which is the lowest level of evidence, in EBM. Therefore, the authors need to limit the question to the value of animal research in clinical medicine and not in EBM. Most clinicians do not use much of bench research or animal research into their clinical decision making based on EBM.*

Response: You raise a valid point about the classification of bench research within the hierarchy of evidence in evidence-based medicine (EBM). It’s important to clarify that while animal models can provide valuable preliminary insights, they often fall short in terms of direct applicability to clinical practice. We made the following changes in the manuscript:

(on page 3) In this framework, levels of evidence are crucial for prioritizing research findings. Evidence is typically ranked in a hierarchy, with systematic reviews and meta-analyses of randomized controlled trials (RCTs) at the top, followed by individual RCTs, cohort studies, and case-control studies. While higher-level evidence is generally considered more reliable, it is crucial that it not be accepted uncritically. Conversely, lower-level evidence, such as findings from animal studies, should not be dismissed outright but interpreted with care, considering the context and limitations inherent to each type of research.

(on page 4) However, animal studies, typically regarded as preclinical evidence, occupy the lowest tier in the evidence hierarchy. Although they provide valuable insights, especially in the early stages of scientific inquiry, their translational relevance to human disease is often limited by significant species differences^[1]^. A growing body of research highlights the disconnect between preclinical results and human outcomes, underscoring the limitations of animal models as reliable predictors of human disease. For instance, in a systematic review, Henderson et al. (Henderson u. a. 2013) identified 26 guidelines and 55 recommendations for designing and executing preclinical efficacy studies to address causal inference threats.

On page 5: The issue of translating preclinical findings into human treatments is particularly evident in cancer research. Despite promising preclinical results, fewer than 15% of clinical trials successfully progress beyond Phase I (Hackam und Redelmeier 2006), with cancer research consistently exhibiting the highest rates of failure (Gamble und Matthews 2010). Even when murine models show initial promise, the success rate of translating these findings into human treatments is less than 8% (Hackam und Redelmeier 2006). This low success rate can be attributed to the extreme complexity of human carcinogenesis, physiology, and disease progression. The difficulty in translating findings from animal models to human systems underscores the inherent limitations of these models in providing reliable evidence for clinical practice.

Moreover, the rise of personalized medicine (PM) has introduced new complexities in assessing treatment efficacy. PM seeks to tailor treatments to individual patients based on specific genetic, molecular, or biomarker profiles (Djulbegovic und Guyatt 2017). While this approach offers the promise of more effective, individualized therapies, it challenges traditional methods of evidence generation, such as RCTs and large cohort studies, which may be less applicable when patient populations are small and highly specific. As PM emphasizes the variability of individual responses to treatment, it raises critical questions about the continued relevance of traditional research models like animal studies in the context of modern, personalized approaches to medicine (Vogt und Hofmann 2022).

This context prompts a pressing question: If animal studies are considered the lowest level of evidence, with at least six higher levels available, and if current technological innovations offer viable alternatives to animal use in various stages of research, why do we continue to rely so heavily on animals in scientific inquiry?

*2. The paper convincingly argues that animal models are often unreliable for translational research due to discrepancies between animal and human biology. However, it could benefit from a more balanced discussion that acknowledges cases where animal models have been crucial in drug development or understanding of physiology and pathophysiology of diseases.  Science and medicine took long time and incremental steps to understand diseases that may not have been possible without the use of animals.*

Response: we did initially structure the paper in a way that the many positive achievements were discussed before moving to the criticism; however, we cut that out in order to narrow the focus. However, in response to the first reviewer’s comment, we have made the following changes to the manuscript:

Animal experimentation has historically played a pivotal role in medical progress, facilitating groundbreaking discoveries such as the germ theory of disease and advancing our understanding of conditions like tuberculosis and cholera. Pioneers like Louis Pasteur (1822-1895) and Robert Koch (1843-1910) relied on animal models to validate their theories and refine medical treatments (Anita Guerrini 2022). The mechanistic view of animals championed by René Descartes during the Renaissance further encouraged scientific experimentation, although it also sparked enduring ethical debates about animal suffering and consciousness (Descartes, 1989; Bentham, 1823). Over time, rodents, particularly mice and rats, became the preferred models due to their small size, rapid reproduction, and low maintenance costs, making them ideal subjects for research (Baumans 2007). In cancer research, Knowledge gained from animal research continues to enhance the well-being of not only humans but also domestic, pet, and wild animals (Kojima, Seidle, und Spielmann 2019). The discovery of antibiotics such as penicillin, erythromycin, and tetracycline would not have been possible without the involvement of animal testing. These advancements highlight the critical role that animal research plays in developing medical treatments that benefit a wide range of species.

*3. The ethical discussion around animal experimentation is robust and provides multiple philosophical frameworks, such as utilitarianism, deontology, and contractarianism. While the ethical perspective is well-argued, it alone might not be sufficient to make the case for or against animal research. The authors could enhance the ethical discussion by integrating more real-world examples of how these ethical considerations have shaped policy or led to alternatives like organoids.*

Response: thank you for the comment. As this comment aligns with the 2^nd^ reviewer critic on limited sources for building a narrative review, the second section “Ethical Perspective: Evidence and Animal” is completely rewritten using other sources than Olsson’s (in response to the 2^nd^ reviewer) and more examples incorporated within the subsections (in response to the 1^st^ reviewer) .

*4. The manuscript is focused on cancer research, a field where pharmacogenomics and precision medicine are well-advanced. While it touches on the potential of personalized approaches in other diseases, it does not provide sufficient evidence to show that the conclusions drawn in oncology would be as applicable in other fields of medicine. Expanding this discussion could strengthen the manuscript's relevance to a broader medical audience especially that the specificity required in PM and cancer treatment does not easily translate to other multifactorial diseases.*

Response: it is indeed a valid point, thank you. The fact that we have focused on cancer, which as you point out, is advanced, is to show that even in cases such as cancer where the knowledge seems to be there, the translation is not entirely successful. Although we would like to focus on one example rather than providing a broader one in the interest of a more focused study, in our response to the comments provided by the reviewers, we have broadened the scope in most of the parts. Additionally, the following (more general) lines are added to the text:

Researchers (Landis et al., 2012) report the same pattern of unreliability and unreplicability in spinal cord injury, stroke, Parkinson’s disease and multiple sclerosis. Furthermore, in cases like Alzheimer’s disease, over 99% of clinical trials fail, with no animal model reliably predicting the clinical effectiveness of Alzheimer’s treatments (Veening-Griffioen et al., 2019) .

*5. Although the authors appropriately focus about the need to minimize animal suffering within the three Rs, they fail to recognize that institutional and funding research committees have significantly evolved over the years in requiring strict protocols in animal humane handling and reduction of unnecessary suffering.*

Response: the following section is added.

Since the introduction of the 3Rs, numerous initiatives have been established to promote alternative research methods. In 1969, the Fund for the Replacement of Animals in Medical Experiments (FRAME) was founded, followed by the journal Alternatives to Laboratory Animals (ATLA). In 1981, the Center for Alternatives to Animal Testing (CAAT) was created at Johns Hopkins University, and the European Reference Laboratory for Alternatives to Animal Testing (EURL-ECVAM) was founded in 1991 to enhance the acceptance of non-animal methods. In 2000, the U.S. Interagency Coordinating Committee on the Validation of Alternative Methods (ICCVAM) was established to validate alternative toxicological tests. The Council for International Organizations of Medical Sciences (CIOMS) published updated guiding principles in 2012, and the ISO 10993-2 standard was introduced in 1996 to incorporate animal welfare. Additionally, the World Animal Health Organization (OIE) included animal welfare standards in its Terrestrial Animal Health Code. These initiatives reflect a global shift towards reducing animal testing and advancing humane research practices (Kurosawa 2019).

Significant progress has already been made by the scientific community, industry, and regulatory bodies in this area. The novel approaches discussed—incorporating cutting-edge computational technologies, bioengineering innovations, and big data analytics—are not entirely new. These methods are actively being developed to reduce the reliance on animal models in cancer research and treatment development, and are increasingly being integrated into mainstream cancer care strategies. Key examples include AI-driven cancer models for drug discovery (You et al., 2022), human microbiome models in cancer research (Kandalai et al., 2023), patient-derived tumor organoids (Yang et al., 2018), crowdsourced cancer data for virtual trials (Lee et al., 2017), CRISPR-based humanized models for targeted cancer therapy (Chehelgerdi et al., 2024), microfluidic-based screening (Sibbits et al., 2024), synthetic biology for cancer research (Zhu et al., 2024), and decentralized clinical trials using blockchain technology (Zhuang et al., 2020). These approaches represent a convergence of interdisciplinary strategies designed to create more accurate, human-relevant, and ethical models for cancer research, thereby advancing the broader goal of animal reduction.

The European Union's ban on animal testing for cosmetics (effective from 2013) exemplifies a deontological response to the ethical concerns raised by animal rights activists. The ban reflects a growing societal recognition that animals should not be subjected to unnecessary suffering for non-essential purposes, such as the production of cosmetic products.

That said, operationalizing these advances remains a significant challenge. Despite the myriad promising alternatives that adhere to the 3Rs principles, we are still, to some degree, dependent on animal studies, particularly in certain stages of research or in contexts where human-relevant models have not yet fully matured (Webster 2022).

*6. Although it is strongly recommended to "focus on patient-centered outcomes" in clinical trials as the authors suggest, this fact is beyond the focus of this paper which is about preclinical animal translation research, and therefore may not be necessary in the conclusion.*

Response: You are absolutely right. This is deleted.

In summary, the manuscript is thorough in its literature review and ethical arguments, but it could benefit from a more balanced discussion of the benefits of animal research, the broader applicability of precision medicine beyond cancer, the current advances in implementing ethical concepts in animal handling, and the practical limitations of implementing alternative technologies.

We hope that we were able to address the issues raised by the first reviewer with our adjustments.

Reviewer #2:
The paper "Lost in translation?" Animal research in the era of precision medicine" analyzes very important ethical and epistemological issues in translational medicine. The authors reviews epistemological perspectives, ethical arguments and then seem to consider these in the context of Cancer Care. Although I think that there are some ideas that are publishable, I do not believe this article is ready for publication at this time for the following reasons:

*1****.*** *Lack of methodological details: The paper seems to be in the "review" section which would require a method used to review the issue. This could be as simple as a narrative literature review. This issue becomes apparent in the section on ethical perspectives on p.5. In this section the authors focus on Olsson and colleagues (11) work to summarize the ethical issues in animal research. This work is more than 20 years old and is more of a handbook for students than a novel or systematic work. There is therefore a substantial lack of research about animal ethics and moral theory. The discussion stays very superficial and only considers main sources through Olsson's lens.*

Response: thank you for your comment. To address this issue, the second section “2. Ethical Perspective: Evidence and Animal” is completely rewritten using other sources than Olsson’s.

2***.*** *Lack of scope: The paper seems to waiver between wanting to make a general argument about animal use to providing a justification to limit animal use in cancer research. The former is not novel and the second would require more work. I do think that a paper explaining how to cut down on animal use in cancer work may help scope the article. One could argue that the ethical and epistemological reasons to reduce animals are clear. However, its operationalization is challenging. If the paper focused on operationalization of animal reduction in cancer care it may yield a better scope.*

Response: This is a highly valuable comment, particularly in shifting the focus to the operationalization of animal reduction in cancer care. However, on the one hand, as the reviewers have pointed out, significant progress has already been made by the scientific community, industry, and regulatory bodies in this area. On the other hand, we believe that the issue with operationalization is connected to the ethical and epistemological aspect. Furthermore, even if the ethics and epistemology is clear for evidence based medicine, precision medicine demands a new perspective.

As is now written in the manuscript (in response to point 5 of reviewer#1) the novel approaches discussed_ incorporating cutting-edge computational technologies, bioengineering innovations, and big data analytics_ are not entirely new. These methods are actively being developed to reduce the reliance on animal models in cancer research and treatment development, and are increasingly being integrated into mainstream cancer care strategies. Key examples include AI-driven cancer models for drug discovery (You et al., 2022), human microbiome models in cancer research (Kandalai et al., 2023), patient-derived tumor organoids (Yang et al., 2018), crowdsourced cancer data for virtual trials (Lee et al., 2017), CRISPR-based humanized models for targeted cancer therapy (Chehelgerdi et al., 2024), microfluidic-based screening (Sibbits et al., 2024), synthetic biology for cancer research (Zhu et al., 2024), and decentralized clinical trials using blockchain technology (Zhuang et al., 2020). These approaches represent a convergence of interdisciplinary strategies designed to create more accurate, human-relevant, and ethical models for cancer research, thereby advancing the broader goal of animal reduction.

That said, as the reviewer astutely points out, operationalizing these advances remains a significant challenge. Despite the myriad promising alternatives that adhere to the 3Rs principles, we are still, to some degree, dependent on animal studies, particularly in certain stages of research or in contexts where human-relevant models have not yet fully matured. We concur with the reviewer’s observation that focusing more explicitly on the operationalization of animal reduction would indeed provide a clearer scope for the paper. However, this would require a substantial shift in focus and scope, as such a discussion would need to address not only technological innovations but also the policy, regulatory, and practical barriers that impede the broader adoption of non-animal models. We have made an effort to address this challenge within the paper, as we recognize the need for more comprehensive work in this area and believe it requires further exploration and clarity. This approach also serves to address the reviewer’s concern about the novelty of the paper, emphasizing that while these ideas are emerging, their true potential for operationalization remains a topic of active and necessary development.

3.      Lack of novelty**:**  The arguments to limit animal use presented are already very well known and not novel. Changing the scope as mentioned previously may help.

Response: In light of your comments, we have considered adjusting the scope of the paper to focus more specifically on the **operationalization** of animal reduction in cancer care. This would allow for a deeper discussion of the **innovative methods** that are emerging, as well as the **challenges** and **opportunities** in translating these approaches into practice. We also see this as an opportunity to address the practical barriers more directly and identify actionable steps toward reducing animal use in cancer research.

Summary of our responses:

We thank the reviewers for taking the time to comment on our manuscript. The comments have been very constructive and have helped us to reformulate our thoughts. We have therefore substantially edited the manuscript; most of the changes are not repeated here in the point-by-point response letter and can be followed in the main text in the word file.

Murugadas, Baumans, V. (2007). The welfare of laboratory mice. In E. Kaliste (Hrsg.), *The Welfare of Laboratory Animals* (S. 119–152). Springer Netherlands. https://doi.org/10.1007/978-1-4020-2271-5_7

Blunt, C. (2022). *The Pyramid Schema: The Origins and Impact of Evidence Pyramids*. https://doi.org/10.13140/RG.2.2.16118.88643

Chehelgerdi, M., Chehelgerdi, M., Khorramian-Ghahfarokhi, M., Shafieizadeh, M., Mahmoudi, E., Eskandari, F., Rashidi, M., Arshi, A., & Mokhtari-Farsani, A. (2024). Comprehensive review of CRISPR-based gene editing: Mechanisms, challenges, and applications in cancer therapy. *Molecular Cancer*, *23*(1), 9. https://doi.org/10.1186/s12943-023-01925-5

Kandalai, S., Li, H., Zhang, N., Peng, H., & Zheng, Q. (2023). The human microbiome and cancer: A diagnostic and therapeutic perspective. *Cancer Biology & Therapy*, *24*(1), 2240084. https://doi.org/10.1080/15384047.2023.2240084

Knight, A. (2019). *Chapter 14 Critically Evaluating Animal Research*. Brill. https://doi.org/10.1163/9789004391192_015

Kojima, H., Seidle, T., & Spielmann, H. (2019). *Alternatives to Animal Testing Proceedings of Asian Congress 2016: Proceedings of Asian Congress 2016*. https://doi.org/10.1007/978-981-13-2447-5

Landis, S. C., Amara, S. G., Asadullah, K., Austin, C. P., Blumenstein, R., Bradley, E. W., Crystal, R. G., Darnell, R. B., Ferrante, R. J., Fillit, H., Finkelstein, R., Fisher, M., Gendelman, H. E., Golub, R. M., Goudreau, J. L., Gross, R. A., Gubitz, A. K., Hesterlee, S. E., Howells, D. W., … Silberberg, S. D. (2012). A call for transparent reporting to optimize the predictive value of preclinical research. *Nature*, *490*(7419), 187–191. https://doi.org/10.1038/nature11556

Lee, Y. J., Arida, J. A., & Donovan, H. S. (2017). The application of crowdsourcing approaches to cancer research: A systematic review. *Cancer Medicine*, *6*(11), 2595. https://doi.org/10.1002/cam4.1165

Morse, H. C. (2007). Building a Better Mouse: One Hundred Years of Genetics and Biology. *The Mouse in Biomedical Research*, *1*, 1–11. https://doi.org/10.1016/B978-012369454-6/50013-3

Sibbits, J., Di Pietro, L., Privitera, A., Cardaci, V., Maugeri, S., Camarda, M., & Caruso, G. (2024). 3D Bioprinting and Microfluidic-Based Devices for Cancer Detection and Drug Treatment: Focus on Prostate Cancer. *Current Medicinal Chemistry*. https://doi.org/10.2174/0109298673298382240307040239

Veening-Griffioen, D. H., Ferreira, G. S., van Meer, P. J. K., Boon, W. P. C., Gispen-de Wied, C. C., Moors, E. H. M., & Schellekens, H. (2019). Are some animal models more equal than others? A case study on the translational value of animal models of efficacy for Alzheimer’s disease. *European Journal of Pharmacology*, *859*, 172524. https://doi.org/10.1016/j.ejphar.2019.172524

Yang, H., Sun, L., Liu, M., & Mao, Y. (2018). Patient-derived organoids: A promising model for personalized cancer treatment. *Gastroenterology Report*, *6*(4), 243. https://doi.org/10.1093/gastro/goy040

You, Y., Lai, X., Pan, Y., Zheng, H., Vera, J., Liu, S., Deng, S., & Zhang, L. (2022). Artificial intelligence in cancer target identification and drug discovery. *Signal Transduction and Targeted Therapy*, *7*(1), 1–24. https://doi.org/10.1038/s41392-022-00994-0

Zhu, B., Yin, H., Zhang, D., Zhang, M., Chao, X., Scimeca, L., & Wu, M.-R. (2024). Synthetic biology approaches for improving the specificity and efficacy of cancer immunotherapy. *Cellular & Molecular Immunology*, *21*(5), 436–447. https://doi.org/10.1038/s41423-024-01153-x

Zhuang, Y., Sheets, L. R., Shae, Z., Chen, Y.-W., Tsai, J. J. P., & Shyu, C.-R. (2020). Applying Blockchain Technology to Enhance Clinical Trial Recruitment. *AMIA Annual Symposium Proceedings*, *2019*, 1276.

1. For a critic on how the mere Pyramid-shaped l representation of evidence see Blunt (Blunt 2022) who has reviewed the evidence pyramid, ist origins and impact on the meaning of evidence.
